# Epigenetics-based treatment strategies for Alzheimer’s disease

**DOI:** 10.18632/aging.204096

**Published:** 2022-05-20

**Authors:** Qing Cao, Wei Wang, Zhen Yan

**Affiliations:** 1 Department of Physiology and Biophysics, State University of New York at Buffalo, Buffalo, NY 14203, USA

**Keywords:** Alzheimer’s disease, epigenetics, histone methylation, EHMT, prefrontal cortex

Alzheimer’s disease (AD) is a progressive neurodegenerative disease characterized by the impairment of cognitive function. While the pathological hallmarks of AD, amyloid plaques and neurofibrillary tangles, have been studied intensively, effective treatment for AD is still lacking. Emerging evidence suggests that gene transcriptional changes due to epigenetic abnormality during aging may play a key role in AD pathophysiology [1].

One of the major epigenetic mechanisms in gene regulation is histone modification, including histone methylation and acetylation. A new study [2] has found the increased histone H3 dimethylation at lysine 9 (H3K9me2, a repressive histone mark) and its catalyzing enzyme euchromatic histone-lysine N-methyltransferase 2 (EHMT2) in prefrontal cortex (PFC) of the P301S Tau AD mouse model. Consistently, the elevated H3K9me2 and EHMT1/2, as well as the excessive suppression of glutamate receptor genes, are also found in PFC of a familial AD model with APP/PS1 mutations [3]. It provides a potential mechanism underlying the diminished expression of synaptic genes that are important for cognitive stability in AD patients uncovered by bioinformatics analyses [4]. A short treatment with the EHMT inhibitor UNC0642 not only ameliorates synaptic and behavioral deficits in both AD models [2,3], but also significantly reduces hyperphosphorylated tau in the P301S Tau AD mouse model [2]. Transcriptomic analysis reveals that a large number of downregulated genes, which are enriched in synaptic organization and plasticity, is restored by UNC0642 treatment in both AD models [2,3]. These studies highlight the therapeutic potential of targeting epigenetic enzymes to normalize downregulated genes in AD [5].

In addition to the downregulation of synaptic genes in AD [4], many genes linked to inflammation, cell stress, DNA damage and apoptosis are upregulated in AD [6]. Another recent study [7] has provided a potential epigenetic mechanism for the abnormal elevation of genes that are harmful to neuronal survival and cognitive function. The authors have found that histone H3 trimethylation at lysine 4 (H3K4me3, a permissive histone mark) is significantly increased in AD patients and AD mouse models. Treatment with an epigenetic compound to inhibit H3K4me3-catalyzing enzymes significantly improves synaptic function and cognitive behaviors. Serum and glucocorticoid-regulated kinase 1 (Sgk1), a multifunctional kinase that plays an important role in cellular stress response [8], is identified as a top-ranking gene target resulting from the elevated H3K4me3 in AD, and a short treatment with Sgk1 inhibitor also ameliorates synaptic and behavioral deficits in the P301 Tau AD model [7]. It highlights the therapeutic potential of targeting epigenetic enzymes to normalize upregulated genes in AD [5].

These findings have provided an important framework for understanding the role of epigenetic dysregulation of distinct sets of genes in AD pathophysiology and suggested a novel epigenetics-based therapeutic strategy for AD. The advantages of epigenetic therapies include: (1) Specific molecules can be targeted to modify different components of the epigenetic machinery; (2) A network of genes can be collectively adjusted to restore cells to their normal state. However, the broad effects of epigenetic regulation may induce wide-spread and off-target consequences. Future studies need to discover more about interactions between the epigenome, the genome and the environment, find cell type-specific epigenetic changes in disease conditions, and develop epigenetic compounds with higher specificity, less side effects and better brain permeability.
